# In search for a standard of rationality

**DOI:** 10.3389/fpsyg.2014.00049

**Published:** 2014-01-30

**Authors:** Emmanuel M. Pothos, Jerome R. Busemeyer

**Affiliations:** ^1^Department of Psychology, City University LondonLondon, UK; ^2^Department of Psychological and Brain Sciences, Indiana UniversityBloomington, IN, USA

**Keywords:** decision making, classical probability theory, quantum probability theory, rationality, conjunction fallacy

The debate on human rationality goes to the heart of fundamental questions about human existence. When can we say that a decision is correct? What is the basis for the achievements of the human intellect? What is the most important cognitive distinction between humans and non-human organisms? Proposals of rationality have an interesting status as psychological theories. They are not quite theories of decision making in practice—such theories are referred to as descriptive theories, to imply that they describe what goes on. Rather, proposals of rationality are normative theories, to imply theories of how people ought to reason, if they seek decision outcomes, which are deemed to be correct, on the basis of some absolute standard (here, we are simplifying a complex debate; arguments have been expressed against a distinction between normative and descriptive rationality as we make above, e.g., Elqayam and Evans, 2011, 2013). Of course, a normative theory must be partly a descriptive theory as well, since it is assumed that humans can, in principle, sometimes, reason on the basis of the normative prescription (they may just not do so, in typical situations, perhaps due to process demands or time or other constraints).

Currently, the dominant approach to normative rationality is based on classical probability (CP) theory. This approach was established after a major shift in conceptual thinking about rationality. Before (effectively since antiquity), it was believed that the standard for correctness in decision making was classical logic. But this position came under intense scrutiny, with experimental results showing that naïve participants, even in simple tasks, would not reason in a way consistent with classical logic (Wason, 1960). One reaction to such results was to develop dual theories of reasoning, which would, broadly speaking, involve a rational component and a heuristics one (cf. Sloman, 1996). However, a priori arguments emerged against any kind of role of logic in human practical decision making (i.e., decision making exempting mathematical/ scientific etc.; Chater and Oaksford, 1993). By contrast, a theory of rationality (and decision making) based on probability theory seemed (and seems) to align itself closely with the intuition we have about what it means for decision making to be successful. Such a theory is about the use of available information from the environment, so as to optimally predict the probability of future events (Anderson, 1991; Oaksford and Chater, 2007; Tenenbaum et al., 2011). The fact that the classical prescription appears to be consistent with human cognition in many cases (e.g., Griffiths et al., 2010, and the above references) also corroborates the psychological relevance of CP theory.

There are formal arguments to support the notion that CP theory provides a *correct* association of probabilities to uncertain events. The Dutch Book Theorem (DBT; e.g., Howson and Urbach, 1993) shows that if one assigns probabilities to events in a way inconsistent with the axioms of CP theory, then it is possible to identify a combination of stakes (money to be won or lost, depending on whether the events occur or not), which guarantees a loss (or gain, depending on the sign of the stakes). That is, according to the DBT, when failing to follow the rules of CP theory, you may be vulnerable to a sure loss (extensions to the DBT, such as the Converse DBT, have been presented too; Vineberg, 2011). Note that the DBT is based on value maximization, but it is well established that reasoners are typically e.g., risk averse (Kahneman and Tversky, 1979). Wakker (2010) showed that risk averse decision makers are subject to a Dutch Book, which provides an interesting conundrum, since expected utility theory, which allows for a risk averse utility function, is considered the rational theory of risky decision making. Nevertheless, the utility of the DBT, in relation to a theory of rationality based on CP theory, is that it provides a formal justification for why CP theory provides the normative prescription for decision making. In other words, currently, if one is interested in whether a probabilistic decision is correct or not, then one needs to explore its consistency with the prescription from CP theory.

The above is an extremely powerful and useful conclusion. Unfortunately, we believe it is vulnerable to criticism. We present two arguments against it, motivated from the interest in applying quantum probability (QP) theory in cognitive modeling. By QP theory, we imply the mathematics for assigning probabilities to events from quantum mechanics, without the physics. QP theory is a formal theory of probability, like CP theory. QP and CP theories are based on different axioms and so their predictions can diverge. QP theory is a plausible contender in decision making (and rationality). Recent work has shown that QP principles can provide the basis for simple, constrained models for empirical findings, which have been persistently problematic from a classical perspective, such as order effects on choice (Moore, 2002), the conjunction fallacy (Tversky and Kahneman, 1983), and the disjunction fallacy (Shafir and Tversky, 1992), for example, in Pothos and Busemeyer (2009), Trueblood and Busemeyer (2011), and Wang and Busemeyer (2013). Moreover, QP principles have been successfully applied in other areas of cognition, such as memory (Bruza et al., 2009), perception (Atmanspacher and Filk, 2010), and conceptual combination [Aerts (2009); overviews in Busemeyer and Bruza (2011), Pothos and Busemeyer (2013), Wang et al. (2013)].

The above points attest to the descriptive status of QP theory in cognitive theory, not its normative status. Nevertheless, they motivate a consideration of explanatory concepts from QP theory in psychological debates. Of relevance presently is the idea of incompatibility, in relation to two (or more) questions (or possibilities etc.). According to classical theory, all questions are compatible, which means that the answer to any set of questions can be known concurrently. Following from Tversky and Kahneman's (1983) experiment which led to the finding of the conjunction fallacy, for example, classically, it can be established that Linda is a bank teller at the same time as deciding whether Linda is a feminist. As a result, it is always possible to specify a joint probability distribution for the outcomes of any arbitrary set of questions (this is the principle of unicity; Griffiths, 2003). The intuition that such questions are compatible appears obvious. How could it possibly be otherwise? Yet, in QP theory questions can be compatible or incompatible. In the latter case, certainty about one inexorably causes uncertainty about the other. Thus, resolving the question about whether Linda is a bank teller requires that we are uncertain about whether she is a feminist, and vice versa. Incompatibility means that there is no single sample space against which we can assess all possible questions about a system of interest (such as Linda). Rather, certainty about a particular question creates a novel perspective (sample space), against which the remaining questions can be assessed (these ideas broadly resonate with Evans's, 2006, 2007, “singularity” principle). Equally, incompatibility implies that it is impossible to define a joint probability distribution for the corresponding questions. One can only define a probability for a sequence of two events, which is order dependent.

The above leads us to our first point. We think that the representational requirements from the principle of unicity are cognitively unrealistic. If we imagine a representation space in which all question outcomes are compatible, then, for two questions, each axis corresponds to a particular combination of outcomes (one axis would correspond to the combination that Linda is a bank teller and not a feminist, etc.). For two questions with binary outcomes, we need a four dimensional space. The consideration of each additional binary question increases the dimensionality of the space by a factor of two, so that, for N binary questions, we require 2^*N*^ dimensions. A classical space for just 10 binary questions requires over 1000 dimensions. The CP theory requirements for representational capacity appear too stringent. Another way to look at this issue is that, regardless of the number of questions considered, classically it is always possible to construct a complete joint probability distribution. But, where would the information come from to construct such a joint probability distribution, especially when considering unfamiliar combinations of questions (such as being a bank teller or a feminist)? Note, the principle of indifference cannot provide a general solution to this issue (e.g., Gilboa, 2009).

Thus, we suggest that cognitively it is more plausible to consider some questions, especially ones not typically considered together, as incompatible. Indeed, there have been suggestions that, with practice, some of the decision making fallacies attenuate (Nilsson et al., 2014; Trueblood, pers. commu.). This conclusion reduces the plausibility that CP theory provides a good descriptive framework for decision making. By implication, QP theory is perhaps a framework for bounded rationality Simon, 1955: Perhaps not as rational as in principle possible (assuming CP theory is the ultimate standard of rationality), but the best that can be achieved, given (broadly assumed) limitations in the representational capacity of the cognitive system.

This discussion leads to our second point: exactly what is the evidence that probabilistic inference on the basis of CP theory is as *accurate as possible*? An a priori argument is the DBT. The consistency in probabilistic inference, which is demonstrated with the DBT, perhaps implies accuracy as well (i.e., do CP theory probabilities match empirical data?). Is it possible to prove a version of the DBT for QP theory as well? Superficially, this may appear not to be the case. First, the axioms of CP theory (on the basis of which the DBT is proved) are very different from those of QP theory. Second, verifiably (e.g., Gilio and Over, 2012), a classical decision maker, committing the conjunction fallacy in Tversky and Kahneman's (1983) experiment, is subject to a Dutch Book, that is, it is possible to specify a combination of stakes for the various hypotheses (Linda is a bank teller; Linda is a feminist; Linda is a bank teller and a feminist), which lead to a sure loss (or gain). However, it is possible to express the requirements for the DBT in terms of the fundamental principles of QP theory. Moreover, it is certainly true that if the questions about Linda are compatible (i.e., if we assume all events can be placed within the same sample space), then a Dutch Book is possible. But, if they are incompatible this is no longer necessarily the case, because the probabilities involved are based on different conditions (orders of evaluation). With work in progress, we are formalizing the relevant intuitions, but the idea is that accepting one incompatible outcome for Linda (e.g., that she is feminist) creates a separate sample space for another (e.g., that she is a bank teller).

We return to the question of the accuracy of probabilistic inference, since, ultimately, this must be the standard against which we assess whether CP theory or QP theory provide a better framework for understanding rationality. Our view is this: if all the relevant questions are compatible, then rationality is best understood in terms of CP theory (actually, the predictions between CP theory and QP theory with compatible questions would be identical; but, if all questions are compatible, why consider QP theory?). However, if some of the questions are incompatible, then QP theory will provide more accurate predictions for probabilistic inference. For example, if some questions are incompatible, then order effects may arise in conjunctions (Trueblood and Busemeyer, 2011; Wang and Busemeyer, 2013), while conjunction in CP theory is commutative (order effects can arise classically, but not without e.g., a conditionalization depending on order, which is unlikely to be known a priori). There are many effects of this kind, that is, ways in which the knowledge that two questions are incompatible can lead us to probabilistic predictions divergent from those using CP theory. Thus, the question of whether QP theory is a better or worse standard for rational decision making, compared to CP theory, boils down to whether there are questions which are incompatible or not (cf. Oaksford, 2013). This is an exciting empirical issue.
